# A Comparative Study of the Characteristics of Cross-Linked, Oxidized and Dual-Modified Rice Starches

**DOI:** 10.3390/molecules170910946

**Published:** 2012-09-12

**Authors:** Hua-Xi Xiao, Qin-Lu Lin, Gao-Qiang Liu, Feng-Xiang Yu

**Affiliations:** 1 National Engineering Laboratory for Rice and By-product Further Processing, Central South University of Forestry & Technology, Changsha 410004, China; Email: xiaoxijingjing@163.com; 2 Faculty of Food Science and Engineering, Central South University of Forestry & Technology, Changsha 410004, China; 3 College of Life Science and Technology, Central South University of Forestry & Technology, Changsha 410004, China; 4 Department of Food Science and Technology, Hunan Biological Electromechanical Polytechnic, Changsha 410127, China; Email: yufengxiang_1@163.com

**Keywords:** rice starch, cross-linked starch, oxidized starch

## Abstract

**:** Rice starch was cross-linked with epichlorohydrin (0.3%, w/w, on a dry starch basis) and oxidized with sodium hypochlorite (2.5% w/w), respectively. Two dual-modified rice starch samples (oxidized cross-linked rice starch and cross-linked oxidized rice starch) were obtained by the oxidation of cross-linked rice starch and the cross-linking of oxidized rice starch at the same level of reagents. The physicochemical properties of native rice starch, cross-linked rice starch and oxidized rice starch were also studied parallel with those of the two dual-modified rice starch samples using rapid visco analysis (RVA), differential scanning calorimetry (DSC), dynamic rheometry and scanning electron microscopy (SEM). It was found that the levels of cross-linking and oxidation used in this study did not cause any significant changes in the morphology of rice starch granules. Cross-linked oxidized starch showed lower swelling power (SP) and solubility, and higher paste clarity in comparison with native starch. Cross-linked oxidized rice starch also had the lowest tendency of retrogradation and highest ability to resistant to shear compared with native, cross-linked, oxidized and oxidized cross-linked rice starches. These results suggest that the undesirable properties in native, cross-linked and oxidized rice starch samples could be overcome through dual-modification.

## 1. Introduction

Starch is a common ingredient used widely in many food and non-food applications. However, the application of native starches is often limited because of shortcomings in their physiochemical properties such as low shear resistance and thermal stability, thermal decomposition and high retrogradation tendency [1,2,3]. Consequently most commercial starches employed in either food or industrial applications have been chemically modified to improve their functionality.

Native rice starches have poor freeze-thaw stability, resistance to shear, and fair stability to retrogradation with moderate clarity and soft texture [4]. The performance and properties of starch solutions can be altered through chemical modifications, by oxidation or addition of charged substituents to the polysaccharide backbone such as cross-linking. Cross-linking will reinforce the granules of starch to be more resistant towards acidic media, heat and shearing [4]. A study revealed a decrease in retrogradation rate and increase in gelatinization temperature with cross-linked starch, and these phenomena are related to the reduced mobility of amorphous chains in the starch granule as a result of intermolecular bridges [1].

Oxidized starch is widely used in both food and non-food industries where film formation and adhesion properties are desired. The major application of oxidized starch is as a surface sizing agent and a coating binder in paper industry. The food products where oxidized starch is used are neutral tasting and low-viscosity items such as lemon curd, salad cream and mayonnaise [5]. Oxidized starch is commonly prepared by reacting starch with a specified amount of oxidant under controlled temperature and pH. Several oxidizing agents have been used, but hypochlorite is the most common chemical used for the production of oxidized starch on an industrial scale.

Modified starch meets the functional properties required in food products, but modified starch still does not overcome some undesirable properties. Cross-linking shows a high retrogradation trend. Oxidation significantly decreases the viscosity of starch paste and the ability to resist shear. Therefore, dual-modification is used further to improve the undesirable characteristics of starch. 

However, until now, most of the works have been focused on corn, wheat, tapioca, sago and potato starches [3,6,7,8,9], few studies have been reported about the physicochemical properties of modified rice starch [4,10]. The objective of this study was to prepare the dual-modified rice starches (cross-linked oxidized starch and oxidized cross-linked starch), and comparatively investigate the physicochemical properties of native unmodified, cross-linked, oxidized and the two kind of dual-modified rice starch samples.

## 2. Results and Discussion

### 2.1. Swelling Power (SP), Solubility and Paste Clarity of Starches

The SP, solubility and paste clarity of native and modified rice starches are presented in Table 1. The SP of native rice starch was 10.1 g/g. After modification, cross-linked starch had the highest SP of 13.9 g/g, and oxidized starch showed the lowest SP of 0.6 g/g. Oxidized cross-linked starch showed a higher SP of 11.6 g/g, and cross-linked oxidized starch showed a lower SP of 9.0 g/g in comparison with native starch. A significant reduction in SP after oxidation was observed. A similar decrease in the SP after oxidation has been reported previously for *Mucuna* bean [11] and normal and waxy corn starches [12]. Leach *et al.* [13] proposed that bonding forces within the granules of starch affected its swelling power. The reduction in the SP after oxidation may be attributed to structural disintegration within the granules of the starch during the modification process. The SP of rice starch showed a significant increase after cross-linking. The bonding forces within the granules of starch were strengthened by the cross-linked reactions, which result in an increase in SP. However, a previous work reported that higher level of cross-linking caused a reduced swelling power. Jyothi *et al.* [14] indicated that SP of epichlorohydrin cross-linked cassava starch decreased with increasing degree of cross-linking. When native and cross-linked starches were oxidized, the cross-linked starch was different towards oxidation from native starch. The degree of cross-linking of cross-linked oxidized starch could be higher compared with cross-linked starch, resulting in a reduced swelling power. In addition, oxidation and cross-linking reagents had an opposite influence on SP of starch granules. The solubility of native starch was 7.2%, cross-linked and cross-linked oxidized starches showed lower solubility, however, oxidized and oxidized cross-linked starches showed higher solubility compared with native starch. The increasing solubility was due to leaching out of the swollen granules on cooking. Cross-links strengthened the structure of starch granules and prevented the starch molecules from leaching out, so less disintegration took place during gelatinization and the solubility was lower. The increase in solubility after oxidation comes as a result of depolymerization and structural weakening of the starch granules [15]. The clarity of starch reflected how light is transmitted through the paste. Compared with the native starch, the paste clarity of cross-linked starch decreased significantly, and the paste clarity of dual-modified starches significantly increased, whereas the paste clarity of oxidized starch increased greatly (Table 1). Paste clarity is the result of rupture of swollen starch granules, and cross-linking improved the integrity of swollen granules, reducing paste clarity [16]. The structural disintegration within the granules of oxidized starch increased paste clarity. The carbonyl and carboxyl functional groups of oxidation in dual-modified starches improved the paste clarity. Paste clarity of cross-linked oxidized starch was higher compared to oxidized cross-linked starch, it may be due to greater functional groups of oxidation in cross-linked oxidized starch. 

**Table 1:** Swelling power, solubility index and paste clarity of native and modified rice starch samples.

Starch samples	Swelling power (g/g)	Solubility (%)	Paste clarity (%T_650_)
Native	10.1 ± 0.3 ^c^	7.2 ± 0.3 ^c^	3.8 ± 0.1 ^d^
Cross-linked	13.9 ± 0.3 ^a^	6.1 ± 0.4 ^d^	1.8 ± 0.07 ^e^
Oxidized	0.6 ± 0.02 ^e^	12.3 ± 0.01 ^a^	86.4 ± 2.3 ^a^
Oxidized cross-linked	11.6 ± 0.4 ^b^	8.5 ± 0.4 ^b^	4.9 ± 0.2 ^c^
Cross-linked oxidized	9.0 ± 0.5 ^d^	4.7 ± 0.3 ^e^	10.6 ± 3.2 ^b^

Data are means of triplicate analyses with standard deviation. Means in the same column with different superscripts were significantly different at the 5% level.

### 2.2. Pasting Properties

The RVA viscosity characteristics of native and modified starches are shown in Table 2. As discussed earlier, the starches cross-linked at a lower reagent concentration of epichlorohydrin (0.25 and 0.5%) showed an increase in the peak and final viscosity. Pasting temperature and paste viscosity of rice starch decreased significantly after oxidation. The reduction following oxidation is a consequence of structural weakening and disintegration during oxidation, and this partially degraded network is not resistant to shearing and cannot maintain the integrity of the starch granule thereby producing a lower viscosity [17]. The decrease in pasting temperature, peak viscosity and breakdown viscosity of cross-linked oxidized starch could be indicative that higher cross-linking has taken place, *i.e*., there were sufficient cross-links to retard the swelling of the starch and cause a decrease in viscosity. As can be observed in Table 2, the setback was related to the rate of retrogradation, in this study, the starch cross-linked at a lower reagent concentration of 0.3% epichlorohydrin was found to be higher. Cross-linking of molecular chains makes the starch granules more ordered and consequently more energy would be required for swelling [18]. After dual-modification of cross-linked oxidation, due to the introduction of carbonyl and carboxyl functional groups, the setback value decreased significantly compared to native, cross-linked and oxidized cross-linked starches. 

**Table 2:** Pasting properties of native, cross-linked, oxidized, oxidized cross-linked and cross-linked oxidized rice starch samples.

Starch samples	Pasting Temp (°C)	Paste viscosity (cP)			
		Peak	Final	Breakdown	Setback
Native	79.2 ± 0.5 ^a^	3096 ± 39 ^c^	3327 ± 40 ^b^	1474 ± 16 ^c^	1705 ± 20 ^b^
Cross-linked	79.2 ± 0.5 ^a^	3346 ± 40 ^a^	3526 ± 43 ^a^	1764 ± 20 ^a^	1944 ± 21 ^a^
Oxidized	72.6 ± 0.4 ^d^	498 ± 7 ^e^	12 ± 2 ^e^	439 ± 6 ^e^	62 ± 2 ^e^
Oxidized cross-linked	77.5 ± 0.4 ^b^	3198 ± 40 ^b^	2152 ± 26 ^c^	1640 ± 18 ^b^	594 ± 8 ^c^
Cross-linked oxidized	75.4 ± 0.3 ^c^	2875 ± 34 ^d^	1968 ± 28 ^d^	1012 ± 14 ^d^	105 ± 3 ^d^

Data are means of triplicate analyses with standard deviation. Means in the same column with different superscripts were significantly different at the 5% level.

### 2.3. Thermal Analysis

The transition temperatures and the enthalpy values of gelatinization of the native and modified rice starch samples observed using the DSC are presented in Table 3. The retrogradation properties of the native and modified rice starch samples after storage at 4 °C for several days are presented in Table 4. Cross-linked starch had higher transition temperatures and enthalpy values of gelatinization and retrogradation compared with native starch, whereas oxidized starch showed lower transition temperatures and the enthalpy values of gelatinization and retrogradation compared with native starch. For the dual-modified starch samples of cross-linking and oxidation, cross-linked oxidized starch showed the lowest trend of retrogradation. The tendency of retrogradation for oxidized cross-linked starch was significantly lower than that of native starch, while significantly higher than that of cross-linked oxidized starch. There was almost no difference between the gelatinization enthalpy values of two dual-modified starch samples.

**Table 3:** Gelatinization properties of native and modified rice starch samples.

Starch samples	*t*_o_ (°C)	*t*_p_ (°C)	Δ *H*_gel_ (J/g)
Native	59.3 ± 0.6 ^c^	87.6 ± 0.6 ^c^	9.84 ± 0.22 ^c^
Cross-linked	83.7 ± 0.8 ^a^	94.6 ± 0.9 ^a^	11.47 ± 0.26 ^a^
Oxidized	57.5 ± 0.4 ^e^	84.1 ± 0.5 ^e^	8.94 ± 0.19 ^d^
Oxidized cross-linked	58.4 ± 0.5 ^d^	86.1 ± 0.5 ^d^	11.04 ± 0.23 ^b^
Cross-linked oxidized	65.6 ± 0.6 ^b^	90.5 ± 0.6 ^b^	11.13 ± 0.25 ^b^

Data are means of triplicate analyses with standard deviation. Means in the same column with different superscripts were significantly different at the 5% level.

**Table 4:** Retrogradation temperatures and enthalpy of gelatinized native and modified rice starch after 7 days’ storage at 4 °C.

Starch samples	*t*_o_ (°C)	*t*_p_ (°C)	Δ *H*_ret_ (J/g)
Native	39.6 ± 0.4 ^c^	50.1 ± 0.5 ^c^	4.6 ± 0.1 ^b^
Cross-linked	42.5 ± 0.5 ^a^	54.2 ± 0.6 ^a^	5.3 ± 0.2 ^a^
Oxidized	34.2 ± 0.1 ^e^	47.5 ± 0.3 ^e^	3.2 ± 0.0 ^d^
Oxidized cross-linked	38.8 ± 0.3 ^d^	49.7 ± 0.5 ^d^	3.9 ± 0.1 ^c^
Cross-linked oxidized	41.6 ± 0.4 ^b^	53.0 ± 0.7 ^b^	2.1 ± 0.0 ^e^

Data are means of triplicate analyses with standard deviation. Means in the same column with different superscripts were significantly different at the 5% level.

Heat of gelatinization reflects the energy required for disrupting the starch granule structure and since cross-linking reinforces the starch granules, more heat will be required for gelatinization [19]. Strengthening the bonding between starch chains through cross-links might have increased the resistance of the granules to swelling leading to higher transition temperature of gelatinization. In contrast, oxidation brought about weakening of starch granules, consequently, less energy was needed to gelatinize the starch granules. Oxidized starch showed lowest transition temperatures (*t*_o_ and *t*_p_) as compared to native, cross-linked and dual-modified starch samples, the decreases in transition temperatures might be due to the weakening of the starch granules, which led to the early rupture of the amylopectin double helices [20]. The Δ*H*_gel_ of dual-modified starch samples (oxidized cross-linked starch and cross-linked oxidized starch) were not significantly different, while the transition temperatures of cross-linked oxidized starch was significantly higher than that of oxidized cross-linked starch, it indicated that at the lower cross-linked level of 0.3% epichlorohydrin, the oxidation reaction occurred in the cross-linked starch, while cross-linked starch was more difficult to oxidize compared with native starch duo to the strengthened structure of starch after cross-linking. The weakened structure of oxidation starch was strengthened through cross-linking. This explained the higher Δ*H*_gel_ of dual-modified starch as compared with native starch. 

Starch gels are metastable and non-equilibrium systems. Therefore, they undergo structural changes during storage [21]. As shown in Table 4, transition temperatures and retrogradation enthalpy (Δ*H*_ret_) at the end of the storage period dropped significantly compared to transition temperatures and Δ*H*_gel_ during gelatinization. The retrogradation enthalpy of all modified starch samples, except cross-linked starch, was lower in comparison to native starch. The retrogradation enthalpy of cross-linked oxidized starch was the lowest. The enthalpy values of the retrograded starch reflect the melting of the crystallites formed by the association between adjacent double helices during gel storage [22]. Retrogradation properties of starch are indirectly influenced by structural arrangements of starch chains within the amorphous and crystalline regions of the ungelatinized granule [8], Amylose aggregation and crystallization were completed within the first few hours of storage while amylopectin aggregation and crystallization occurred during later stages [23]. Cross-linking resulted in ordered structure in the starch pastes, thus resulting in higher degree of retrogradation. The crystallinity of the starch granules was disrupted due to the weakening of structure during oxidation, it explained that oxidized starch showed lower retrogradation value of enthalpy compared to native and cross-linked starch. The introduction of various functional groups in dual-modified starch hindered double helix formation by disrupting the rearrangements of starch chains during storage, resulting in a decreasing in retrogradation enthalpy. The decreasing of retrogradation enthalpy in cross-linked oxidized starch was more significant than in oxidized cross-linked starch. 

### 2.4. Flowing Characteristics

**Figure 1:**
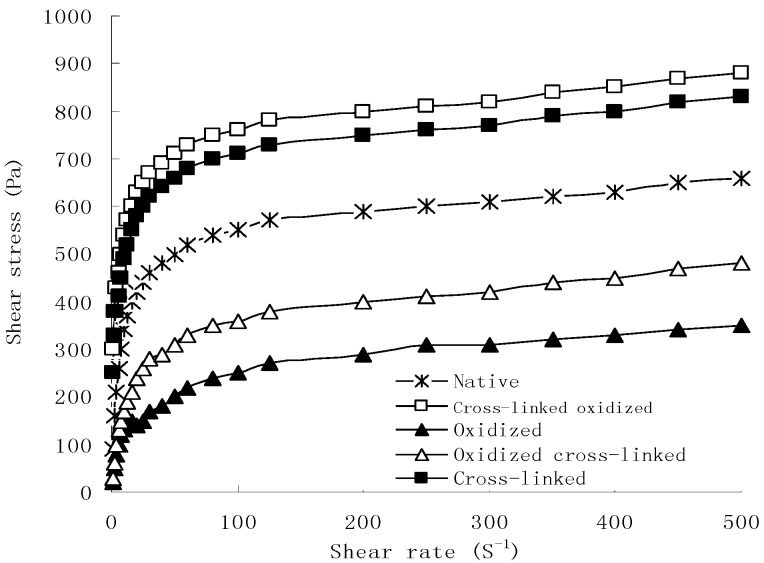
Shear stress as a function of shear rate for native and modified rice starch samples.

The results from the previous studies indicated that rice starch samples were pseudoplastic and shear thinning liquids. As shown by the flow curves in Figure 1, shear stress of all native and modified starch samples increased with increasing shear rate. At the same shear rate, cross-linked starch showed the highest shear stress, while oxidized starch showed the lowest shear stress. Gibinski *et al.* indicated that high values of shear stress pointed to a high stability of the structure of the starch [24]. According to these authors cross-linked starch had the most stable structure and the strongest ability to resist shear, the structure of oxidized starch was weakened and lost the ability to resist shear. Strengthening the bonding between starch chains through cross-links resulted in a stable structure. However, a partially degraded network was not resistant to shear and could not maintain the integrity of starch granules after oxidation, resulting in a decrease in shear stress. Oxidized cross-linked starch showed lower ability to resist shear in comparison to native starch, while it showed higher ability to resist shear in comparison to oxidized starch. Cross-linked oxidized starch showed the highest ability to resist shear of all starch samples. The reaction order of dual-modification had a significant influence on the physicochemical proper of starch granules.

### 2.5. Morphological Characteristics

**Figure 2:**
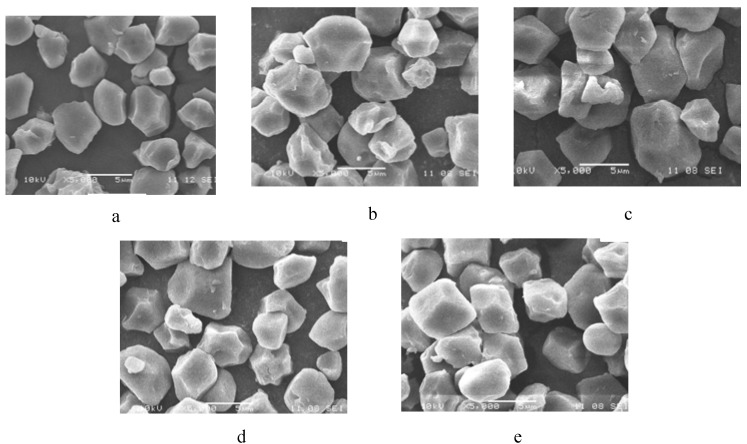
Scanning electron micrographs of native and chemically modified rice starch samples (×5,000; scale bar = 5 μm). (**a**), native; (**b**), cross-linked; (**c**), oxidized; (**d**), oxidized cross-linked; (**e**), cross-linked oxidized.

Scanning electron micrographs of native and chemically modified starch samples at 5000 × magnification are presented in Figure 2. Scanning electron microscopy revealed that starch granules of native and modified rice starch samples were small, and no noticeable differences were observed among the appearances of morphologies of the native and chemically modified starch granules. This indicated that the levels of cross-linking and oxidation used in the present study did not cause any significant changes in the size of rice starch granules. This is consistent with the results reported by Yeh and Yeh [10]. Van Hung and Morita reported that starch granule size had a significant effect on morphological properties of starch granules [25]. Since oxidation brought about weakening of starch granules, it was easy to carry out cross-linking of rice starch after oxidation, and cross-linked oxidized starch showed a lower trend of retrogradation and higher ability to resist shear compared with native and other modified starch samples. 

## 3. Experimental

### 3.1. Materials

Native unmodified rice starch (made from bad mouth-feeling rice) was purchased from Puer Yongji Biological Technology Co. Ltd. (Kunming, China). Sodium hypochlorite containing 5% active chlorine and epichlorohydrin (0.3%, w/w, on a dry starch basis) were purchased from NongHe Co. Ltd. (Changsha, China). All chemicals and reagents used in this study were of analytical grade.

### 3.2. Preparation of Cross-Linked Rice Starch

Cross-linking reaction of starch was performed according to the method of Reddy and Seib [26] with a slight modification. Starch (100 g, dry basis) was suspended in distilled water (150 mL) with addition of NaCl (3 g) and continuously stirred at 25 °C. After adjusting to pH 10.0 with 1 mol/L NaOH, epichlorohydrin (0.3%, w/w, on a dry starch basis) was added directly to the slurry with stirring at 25 °C for 3 h, then adjusted to pH 6.0–6.5 with 0.2 mol/L HCl and the cross-linked rice starch was isolated by centrifugation (3,000 × *g*, 15 min). After washing with distilled water, the sediment was dried at 45 °C for 48 h in a vacuum oven.

### 3.3. Preparation of Oxidized Rice Starch

The oxidized rice starch was prepared by following the method of Autio *et al.* [27] with a slight modification. Starch (100 g, dry basis) was suspended in distilled water (150 mL), and maintained at 30 °C in a heating mantle and the pH was adjusted to 8.5 with 2 mol/L NaOH. Sodium hypochlorite [NaOCl, 2.5 g Cl/100 g starch, 2.5% (w/w)] was slowly added into the starch slurry over 30 min while maintaining the pH 8.5 with 1 mol/L H_2_SO_4_. After the addition of NaOCl, the pH of the slurry was maintained at 8.5 with 1 mol/L NaOH for an additional 30 min. The slurry was then adjusted to pH 6.5–7.0 with 1 mol/L H_2_SO_4_, the oxidized starch was isolated by centrifugation (3,000 × *g*, 15 min), washed with water and dried in a vacuum oven at 45 °C for 48 h.

### 3.4. Preparation of Dual-Modified Rice Starch

Oxidized cross-linked rice starch was prepared by first cross-linking with EPI (0.3%, w/w, on a dry starch basis) as described above. After cross-linking, the pH of the slurry was adjusted to 8.5 with 1 mol/L H_2_SO_4_ and then NaOCl (2.5% active chlorine concentration) was added as previously described. The OCRS was also recovered using the same procedure as for the CRS. Cross-linked oxidized rice starch was prepared by first oxidation with NaOCl (2.5% active chlorine concentration) as described above. After oxidation, the pH of the slurry was adjusting to 10.0 with 1 mol/L NaOH, and then epichlorohydrin (0.3%, w/w, on a dry starch basis) was added as previously described. The cross-linked oxidized rice starch was obtained.

### 3.5. Determination of Swelling Power (SP), Solubility and Paste Clarity

The SP of native and modified starches was measured according to the method of Sasaki and Matsuki [28] with a slight modification. The starch (0.16 g, dry base, db) was suspended in distilled water (5 mL) in glass tubes covered with screw caps. Then the slurry was heated at 90 °C in a shaking water bath for 30 min. After cooling quickly to room temperature in a cold water bath, the samples were centrifuged at 3,000 × *g* for 15 min. The supernatant was decanted carefully and retained, and the SP was determined as weight of the sediment according to: 



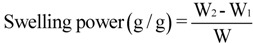



where W_2_ is weight of tube after supernatant was removed (g); W_1_ is weight of dry tube (g) and W is weight of db starch (g). The supernatant was dried at 120 °C for 2 h, the solubility was measured according to:



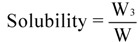



where W_3_ is weight of dried supernatant; W is weight of weight of db starch (g). The paste clarity of native and modified starch was determined based on the method of the Perera and Hoover [29]. For the determination, 1% aqueous suspension of each sample was heated in a water bath at 95 °C for 30 min with constant stirring. The paste was cooled to room temperature. The paste was stored for 24 h in a refrigerator at 4 °C and transmittance was measured at 650 nm against a water blank using a UV-Visible spectrophotometer Model UV 1601 Version 2.40 (Shimadzu, Kyoto, Japan).

### 3.6. Pasting Properties

The pasting properties of rice starch were measured according to our previous method by using a Rapid Visco Analyzer (RVA super 4, Newport Scientific, Sydney, Australia) [30]. Viscosities of starches were recorded with starch suspensions (moisture Content 12.0%, sample 3.00 g, water 25.00 mL) that underwent a controlled heating and cooling cycle under constant shear where the sample was held at 50 °C for 1 min, heated from 50 °C to 95 °C at 5 °C/min and held at 95 °C for 2.7 min, cooled from 95 °C to 50 °C at 5 °C/min and held at 50 °C for 2 min. The initial speed of blender in 10 s was 960 rpm, and after that it was maintained at 160 rpm. Pasting parameters such as pasting temperature, peak viscosity, breakdown (peak viscosity—hot paste viscosity), final viscosity, setback (final viscosity—hot paste viscosity) were recorded. 

### 3.7. Differential Scanning Calorimetry (DSC)

The thermal properties of the samples were carried out using 61 DSC-Pyris Diamond (Perkin-Elmer Corp., Norwalk, CT, USA). Starch samples were weighed into aluminum DSC pans, and deionized water was added by micropipette to achieve a water-sample ratio of 2:1. The sample pans were sealed and allowed to stabilize at room temperature for 24 h before heating. Samples were heated at a rate of 10 °C/min from 25 to 100 °C, using an empty pan as reference. The onset temperature (*t*_o_) and peak temperature (*t*_p_) were determined from the run heating DSC curves. Enthalpy of gelatinization (Δ*H*_gel_) was evaluated based on the area of the main endothermic peak. After the DSC run, gelatinized starch samples were stored at 4 °C for 7 days for retrogradation studies, these stored samples were scanned under the same conditions and the transition temperature and retrogradation enthalpy (Δ*H*_ret_) were determined from the second heating run. 

### 3.8. Measurement of Flow Behavior

Starch paste (8%) was put into the testing platform of dynamic rheometer (ARES, TA, Ltd., New Castle, DE, USA). Viscometry was performed using a controlled strain rheometer using a parallel plate mould (40 mm diameter and 1 mm gap). After trimming off the over-loaded portion of samples around plates, the open side of samples was covered with a thin layer of silicon oil to prevent moisture loss. Shear stress with increasing shear rate (0–500 s^−1^) was obtained at 20 °C to characterize flow behavior.

### 3.9. Morphological Properties

Scanning electron micrographs of native and chemically modified starch samples were obtained using a scanning electron microscope (Model JSM-6380-LV, JEOL, Tokyo Japan) at an accelerating voltage of 10 KV. Starch granules were sprinkled onto double-backed cellophane tape attached to a stub before coating with gold-palladium.

### 3.10. Statistical Analyses

The data reported in the tables were average of triplicate observations. Data obtained were analyzed by single factor analyses of variance (ANOVA) using SPSS for Windows version 13.0. Confidence interval of sample means was reported at the 95% confidence probability. Comparisons of means were made using least significant difference (LSD) and shortest significant ranges (SSR) at 5% significance level (*p* < 0.05). 

## 4. Conclusions

Scanning electron micrographs of native and modified rice starch samples indicated that chemical modification of cross-linking and oxidation had no significant effect on the morphological properties of rice starch granules. At a cross-linking level of 0.3% epichlorohydrin, the obtained cross-linked starch was more difficult to oxidize compared to native starch. The low degree of cross-linking results in an increase in swelling power and solubility, and a decrease in paste clarity, however, a higher degree of cross-linking results in a decrease in swelling power and solubility, and an increase in paste clarity. Oxidation reduced the swelling power, pasting temperature and paste viscosity, and increased the solubility and paste clarity. Cross-linking increased the retrogradation tendency of starch, while oxidation decreased the retrogradation tendency of starch. The tendency of retrogradation of cross-linked oxidized rice starch was the lowest in all native and modified starch samples. Meanwhile cross-linked oxidized starch showed the highest ability to resist shear among all tested starch samples. It can thus be deduced that high quality modified rice starch, cross-linked oxidized starch was obtained by dual-modification, and it can more effectively meet the functional properties demands for food products. Further studies are required to elucidate the detailed structural changes in rice starch during the process of cross-linking and oxidation.

## References

[1] Singh J., Kaur L., McCarthy O.J. (2007). Factors influencing the physico-chemical, morphological, thermal and rheological properties of some chemically modified starches for food applications—A review. Food Hydrocolloid..

[2] Kaur M., Oberoi D.P.S., Sogi D.S., Gill B.S. (2011). Physico-chemical, morphological and pasting properties of acid treated starches from different botanical sources. J. Food Sci. Technol..

[3] Wang Y.J., Wang L. (2003). Physicochemical properties of common and waxy corn starches oxidized by different levels of sodium hypochlorite. Carbohydr. Polym..

[4] Raina C.S., Singh S., Bawa A.S., Saxena D.C. (2006). Some characteristics of acetylated, cross-linked and dual modified Indian rice starches. Eur. Food Res. Technol..

[5] Lawal O.S. (2004). Composition, physicochemical properties and retrogradation characteristics of native, oxidized, acetylated and acid-thinned new cocoyam (*Xanthosoma sagittifolium*) starch. Food Chem..

[6] Ačkar D., Šubarić D., Babić J., Miličević B., Jozinović A. (2012). Modification of wheat starch with succinic acid/acetanhydride and azelaic acid/acetanhydride mixtures. II. Chemical and physical properties. J. Food Sci. Technol..

[7] Atichokudomchai N., Varavinit S. (2003). Characterization and utilization of acid-modified cross-linked Tapioca starch in pharmaceutical tablets. Carbohydr. Polym..

[8] Wattanachant S., Muhammad K., Hashim D.M., Rahman R.A. (2003). Effect of crosslinking reagents and hydroxypropylation levels on dual-modified sago starch properties. Food Chem..

[9] Kaur L., Singh J., Singh N. (2006). Effect of cross-linking on some properties of potato (*Solanum tuberosum* L.) starches. J. Sci. Food Agric..

[10] Yeh A.-I., Yeh S.-L. (1993). Some characteristics of hydroxypropylated and cross-linked rice starch. Cereal Chem..

[11] Adebowale K.O., Lawal O.S. (2003). Functional properties and retrogradation behaviour of native and chemically modified starch of *Mucuna* bean (*Mucuna pruriens*). J.Sci. Food Agric..

[12] Sandhu K.S., Kaur M., Singh N., Lim S.-T. (2008). A comparison of native and oxidized normal and waxy corn starches: Physicochemical, thermal, morphological and pasting properties. LWT-Food Sci. Technol..

[13] Leach H.W., McCowen L.D., Schoch T.J. (1950). Structure of the starch granule. I. Swelling and solubility patterns of various starches. Cereal Chem..

[14] Jyothi A.N., Moorthy S.N., Rajasekharan K.N. (2006). Effect of cross-linking with epichlorohydrin on the properties of cassava (*Manihot esculenta* crantz) starch. Starch.

[15] Hodge J.E., Osman E.M., Fennema O.R. (1996). Carbohydrates. Food Chemistry.

[16] Zheng G.H., Han H.L., Bhatty R.S. (1999). Functional properties of cross-linked and hydroxipropylated waxy hull-less barley starches. Cereal Chem..

[17] Morton M.W., Solarek D., Whistler R.L., BeMiller J.N., Paschall E.F. (1984). Starch derivatives: Production and Uses. Starch Chemistry and Technology.

[18] Kartha K.P.R., Srivastava H.C. (1985). Reaction of epichlorohydrin with carbohydrate polymers. Starch.

[19] Liu H., Ramsden L., Corke H. (1999). Physical properties of cross-linked and acetylated normal and waxy rice starch. Starch.

[20] Adebowale K.O., Afolabi T.A., Lawal O.S. (2002). Isolation, chemical modification and physicochemical characterization of *Bambarra groundut* (Voandzeia subterranean) starch and flour. Food Chem..

[21] Ferrero C., Martin M.N., Zantzky N.E. (1994). Corn-starch-xanthan gum interaction and its effect on the stability during storage of frozen gelatinized suspensions. Starch.

[22] Hoover R., Senanayake S.P.J.N. (1996). Composition and physicochemical properties of oat starches. Food Res. Int..

[23] Sodhi N.S., Singh N. (2005). Characteristics of acetylated starches prepared using starches separated from different rice starch. J.Food Eng..

[24] Gibinski M., Kowalski S., Sady M., Krawontka J., Tomasik P., Sikora M. (2006). Thickening of sweet and sour sauces with various polysaccharide combinations. J.Food Eng..

[25] Van Hung P., Morita N. (2005). Effect of granule sizes on physicochemical properties of cross-linked and acetylated wheat starches. Starch.

[26] Reddy I., Seib P.A. (2000). Modified waxy wheat starch compared to modified waxy corn starch. J. Cereal Sci..

[27] Autio K., Suortti T., Hamunen A., Poutanen K. (1996). Heat-induced structural changes of acid-hydrolysed and hypochlorite-oxidized barley starches. Carbohydr. Polym..

[28] Sasaki T., Matsuki J. (1998). Effect of wheat starch structure on swelling power. Cereal Chem..

[29] Perera C., Hoover R. (1999). Influence of hydroxypropylation on retrogradation properties of native, defatted and heat-moisture treated potato starches. Food Chem..

[30] Xiao H., Lin Q., Liu G.Q. (2012). Effect of cross-linking and enzymatic hydrolysis composite modification on the properties of Rice Starches. Molecules.

